# Simulated Microgravity Combined with Polyglycolic Acid Scaffold Culture Conditions Improves the Function of Pancreatic Islets

**DOI:** 10.1155/2013/150739

**Published:** 2013-08-06

**Authors:** Yimin Song, Zheng Wei, Chun Song, Shanshan Xie, Jinfa Feng, Jiehou Fan, Zengling Zhang, Yubo Shi

**Affiliations:** ^1^Department of General Surgery, Peking Union Medical College Hospital, Beijing 100730, China; ^2^The Key Laboratory of Cell Transplantation of Ministry of Health and Department of General Surgery, The First Affiliated Hospital of Harbin Medical University, No. 23 Youzheng Street, Nangang District, Harbin, Heilongjiang, China; ^3^General Surgery, Heilongjiang Provincial Hospital, Nangang Branch, Harbin 150001, China

## Abstract

The *in vitro* culture of pancreatic islets reduces their immunogenicity and prolongs their availability for transplantation. Both simulated microgravity (sMG) and a polyglycolic acid scaffold (PGA) are believed to confer advantages to cell culture. Here, we evaluated the effects of sMG combined with a PGA on the viability, insulin-producing activity and morphological alterations of pancreatic islets. Under PGA-sMG conditions, the purity of the islets was ≥85%, and the islets had a higher survival rate and an increased ability to secrete insulin compared with islets cultured alone in the static, sMG, or PGA conditions. In addition, morphological analysis under scanning electron microscopy (SEM) revealed that the PGA-sMG treatment preserved the integral structure of the islets and facilitated islet adhesion to the scaffolds. These results suggest that PGA-sMG coculture has the potential to improve the viability and function of islets *in vitro* and provides a promising method for islet transplantation.

## 1. Introduction 

Diabetes mellitus is characterized by metabolic disorders and abnormally high blood glucose levels, which are caused by the destruction of *β* cells of the pancreas, insulin resistance, and/or insulin deficiency [1, 2]. Pancreatic islet transplantation helps patients with type 1 diabetes by normalizing their glucose metabolism and improving other complications of diabetes [3–6]. However, islets cultured alone *in vitro* exhibit poor viability, a short survival time, and decreased secretory function, mainly due to limited nutrient uptake and waste excretion [7]. 

Recently, both microgravity (MG) and biodegradable polymer scaffolds were confirmed to provide significant advantages in cell culture [8–13] by improving the metabolic microenvironment [14–16]. A bioreactor (rotary cell culture system, RCCS) provides continuous medium rotation and simulates some aspects of microgravity conditions [17, 18], wherein cells suspended in a high aspect ratio vessel (HARV) undergo continuous free fall at a terminal velocity with a low hydrodynamic shear stress force, low turbulence, and a high rate of mass transfer of nutrients [19, 20]. In addition, the HARV is equipped with a semipermeable membrane that permits the diffusion of oxygen and carbon dioxide [21] and provides a better environment for the growth and metabolism of many tissues and cells [22]. Most importantly, the production of IFN-*γ*, IL-1*β*, and TNF-*α* is almost completely abrogated in microgravity cultures [23], significantly decreasing the immunogenicity and restoring the function of secretory cells [24].

Polymers are endowed with basic properties such as biodegradability and biocompatibility [25], and polymers used in medical applications should be nontoxic, nonantigenic, and nonimmunogenic [26, 27]. Additionally, polymer scaffolds may be cut into desired shapes for the growth of various tissues and cells [28] and facilitate the cellular production of an extracellular matrix (ECM). Polymer materials have been approved for use in tissue regeneration and repair [29–31].

In our recent study, a polyglycolic acid (PGA) scaffold reversed a previously observed decrease in islet viability and improved the secretory function of pancreatic islet cells [7]. In this study, we cultured islets under the conditions of stasis, with a PGA scaffold alone, in simulated microgravity (sMG) alone, and with a PGA scaffold combined with sMG (PGA-sMG) and compared the viability, insulin secretory function, and morphology of the islets under these four conditions. 

## 2. Materials and Methods

### 2.1. Animals

Adult female and male Wistar rats, weighing 250 to 300 g, were used in all studies. The tested Wistar rats were supplied by the Animal Care Center of the First Affiliated Hospital of Harbin Medical University. The protocol used in this study was approved by the Animal Ethics Committee of the First Affiliated Hospital of Harbin Medical University.

### 2.2. Isolation and Purification of Islets

Wistar rats that fasted for 12 h were anaesthetized by intraperitoneal injection with 30 mg/kg 0.5% pentobarbital sodium (Shanghai Chemical Reagent Inc., Shanghai, China). After exposing the pancreatic tubes and common bile ducts, 8–10 mL of chilled 0.25% type V collagenase solution (Sigma, St. Louis, Mo, USA) was injected into the pancreas via the pancreatic tubes. After the pancreas was completely expanded, it was removed and placed into a shaking vessel within a water bath for digestion for 10–15 min at 38°C. After the pancreas had been digested into fine particles, 30 mL of chilled Roswell Park Memorial Institute-1640 medium (RPMI-1640 medium, HyClone, Logan, UT, USA) containing 20% fetal bovine serum (FBS, Hyclone), 1% penicillin-streptomycin-amphotericin B (Amresco, USA), and 10 mM Hepes (HyClone) was added to stop the digestion. The solution was incubated at room temperature for 5–10 min and then filtered through a 500 *μ*m stainless steel mesh. The islet cells were purified by the Ficoll method (Pharmacia, Piscataway, NJ, USA). Dithizone (DTZ, Sigma) staining with jacinth enabled the calculation of islet purity as the number of stained cells divided by the total number of cells, and islets of ≥85% purity were selected for use in further experiments.

### 2.3. Preparation of the Porous PGA Fiber Scaffold

Porous PGA (Synthecon, USA) fiber scaffolds were constructed with 13–15 *µ*m diameters and 100–150 *µ*m aperture sizes. The PGA scaffolds were soaked in 75% alcohol for 30 min three times, washed with phosphate-buffered saline, and sterilized under an ultraviolet lamp for 30 min to 1 h. The scaffolds were soaked in 10 mg/mL poly-L-lysine (Mr: 150,000 to 300,000, Sigma) for 30 min and then dried.

### 2.4. Islet Culture

The islets were seeded at a density of [2 × 10^4^] cells per 10 mL HARV (HARV, Synthecon). Control islets were cultured in RPMI-1640 containing 20% fetal bovine serum and 1% penicillin-streptomycin-amphotericin B (Amresco, USA) under static culture conditions. Islets in the sMG group were cultured in HARVs connected to a RCCS (Synthecon). The speed of rotation was gradually increased from 15 to 45 rpm, and air evacuation prevented damage to the islets. The test cell culture contained 10 mg/mL poly-L-lysine-modified sterile PGA scaffolds with islet cells on their surface. For the sMG-PGA co-culture group, islets cultured on PGA scaffolds were placed under sMG conditions. The above-mentioned culture medium was used for the latter three groups. All four groups were cultured under 5% CO_2_ at 37°C.

### 2.5. AO-PI Staining

Islets on the PGA scaffolds were eluted with Hank's solution and collected by centrifugation, and Acridine Orange-Propidium Iodide (AO-PI, Sigma) double fluorescent staining was performed to visualize cell viability. The AO-PI was prepared as follows: 0.01 mL of 670 *μ*mol/L AO and 1 mL of 750 *μ*mol/L PI were mixed together and then diluted 10-fold with Hank's solution. The final solution was sterilized using a 0.22 *µ*m filter. The islets were mixed with filtered AO-PI, incubated for 5–10 min, and then examined by fluorescence microscopy (Olympus, Japan). When viewed under the microscope, the viable cells appeared green (AO stained) and dead cells appeared red (PI stained).

### 2.6. Determination of Islet Viability

A Cell Counting Kit-8 (CCK-8; Dojindo Molecular Technologies, Beijing, China) was used to evaluate cell viability. Cells were seeded at [2 × 10^4^] cells/well in 96-well plates. At different time points, the culture medium was replaced with 100 *μ*L of fresh medium containing 10 *μ*L of the CCK-8 solution. The cells were further incubated for 1.5 h at 37°C, and the optical density (OD) at 450 nm was measured. The mean absorbance at different time points from three separate experiments was plotted with GraphPad Prism software version 5.00 for Windows.

### 2.7. Assessment of Insulin-Producing Activity

The insulin production levels of the islets were measured by radioimmunoassay (Lifescan, USA). A glucose-stimulated insulin-release assay was performed by culturing islets in low-glucose (5.6 mmol/L) RPMI-1640 without FBS for 4 h, after which the culture supernatants were collected for insulin content analysis. The islets were subsequently transferred to high-glucose (16.7 mmol/L) RPMI-1640 without FBS for four hours, and the culture supernatants were collected as before. The insulin levels from the islets incubated in the high-glucose medium were divided by the insulin levels from the islets cultured in the low-glucose medium, and this ratio was used as the insulin-releasing index. The total insulin content of the cells was determined using an Insulin Radioimmunoassay Kit (insulin RIA kit, Linco Research, St. Charles, MO, USA). Briefly, the cells were incubated in 1% hydrochloric acid alcohol at 4°C overnight. After centrifugation, the supernatants were harvested, and the cellular insulin content was analyzed and standardized based on the total intracellular protein content measured with a Bicinchoninic Acid protein assay kit (BCA protein assay kit, Beyotime). The mean concentration of the insulin releasing index or the cellular insulin content from three separate experiments was plotted with GraphPad Prism software version 5.00 for Windows.

### 2.8. Examination under Scanning Electron Microscopy

Scanning electron microscopy (SEM, S-3400N, Hitachi, Japan) was used to examine islet morphology. For the SEM analysis, samples were first fixed with 3% glutaraldehyde for 24 h at 4°C. Then, the samples were washed, fixed with 1% osmic acid for 2 h, and dehydrated with increasing concentrations of 50, 70, and 90% ethanol for 15 min at each concentration, followed by 100% ethanol for 10 min. The samples were frozen, dried, coated, and visualized by**  **scanning electron microscopy. 

### 2.9. Statistical Analysis

The results reported were expressed as the mean values ± standard deviation (SD). Comparisons of different groups were conducted with repeated measurements and at different time points with a one-way analysis of variance (ANOVA) followed by LSD's *t*-test. A *P* value of *P* < 0.05 was considered to be statistically significant.

## 3. Results 

### 3.1. Evaluation of Islet Viability

**Figure 1:**
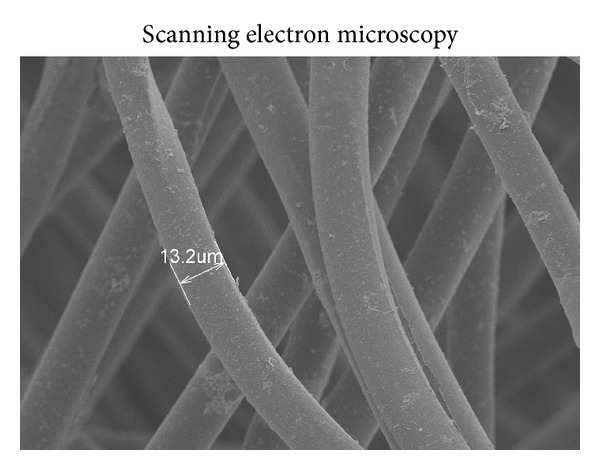
Image of a porous PGA fiber scaffold.

**Figure 2:**
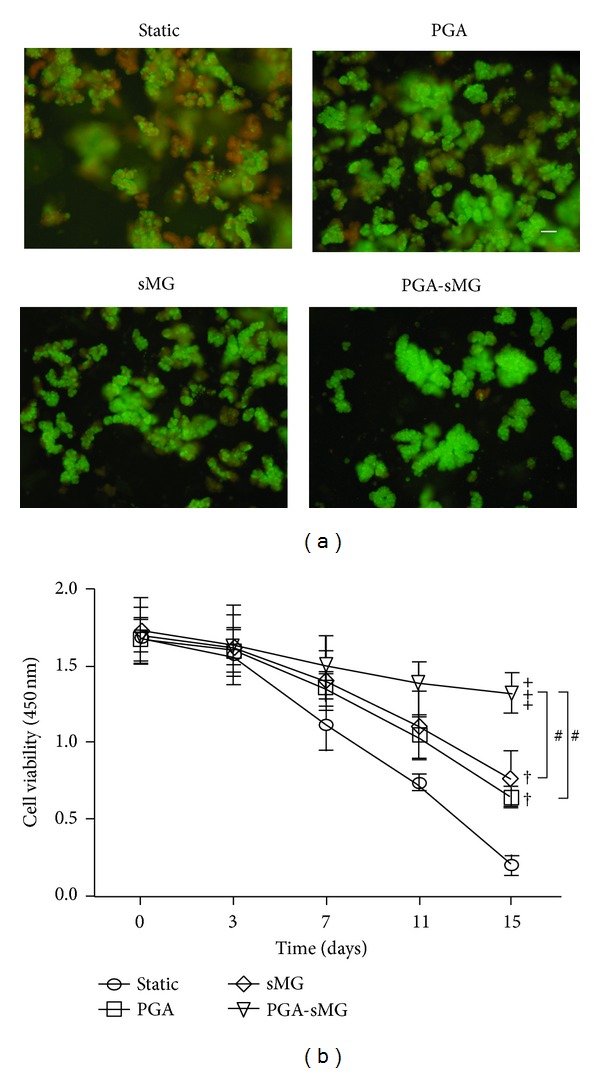
Evaluation of islet viability. (a) AO-PI double fluorescent staining was performed to visualize cell viability. Viable islets are green and dead islets are red (×200) (bar = 100 *µ*m). (b) Islets were cultured and their viability was assessed using a CCK-8 assay. The optical density (OD) at 450 nm was determined at the indicated time points as a measure of cell viability. Significant increases are indicated by “†” at *P* < 0.05, “++” at *P* < 0.01, and “+++” at *P* < 0.001, compared with the static group. A significant difference between PGA-sMG and PGA or sMG is denoted by “#.”

Given the insufficient nutrition provided under 2D culture conditions, PGA scaffolds characterized by excellent biocompatibility and biodegradability were used as carriers for cell culture under 3D conditions. As shown in Figure 1, the PGA scaffolds were microscopically netlike with 13.2 *µ*m diameters. To compare the effects on the cells being cultured alone under static, PGA, and sMG conditions and being cocultured under the PGA-sMG condition, AO-PI staining and colorimetric assays were performed to evaluate cellular viability. As shown in Figure 2(a), cells collected from all groups after culture for 15 days were visualized under fluorescent microscopy. The fluorescent staining demonstrated that there were more viable islets (green) and fewer dead islets (red) in the PGA-sMG group, moderate numbers of viable and dead islets in both the PGA and sMG groups, and fewer viable and more dead islets in the static group.

Subsequently, cellular viability was quantitatively assessed with a CCK-8 kit. As shown in Figure 2(b), this analysis revealed that the survival rates in all groups were time dependent with a gradual decrease. Specifically, at the early time points (days 0 and 3), no measurable alterations with regard to cellular viability were detected in any of the groups. Compared with the matching static condition, the survival rate of cells in the PGA group still displayed no difference in viability at day 7 but increased by 40% (*P* < 0.05) and by more than 2-fold (*P* < 0.01) at days 11 and 15, respectively. Similar alterations in the cellular viability for cells cultured under sMG conditions were observed at days 7, 11 (*P* < 0.05), and 15 (*P* < 0.01). At days 7, 11, and 15, the cellular viability of the PGA-sMG group was 36% (*P* < 0.05), nearly 1-fold (*P* < 0.01) and 6-fold (*P* < 0.001) higher, respectively, than the matching static conditions. After day 11, differences between the PGA-sMG and the sMG or PGA conditions could be detected in their respective cellular viabilities. Interestingly, there was no difference in the survival rate between the PGA and sMG groups at any time point.

### 3.2. Assessment of Insulin-Producing Function

**Figure 3:**
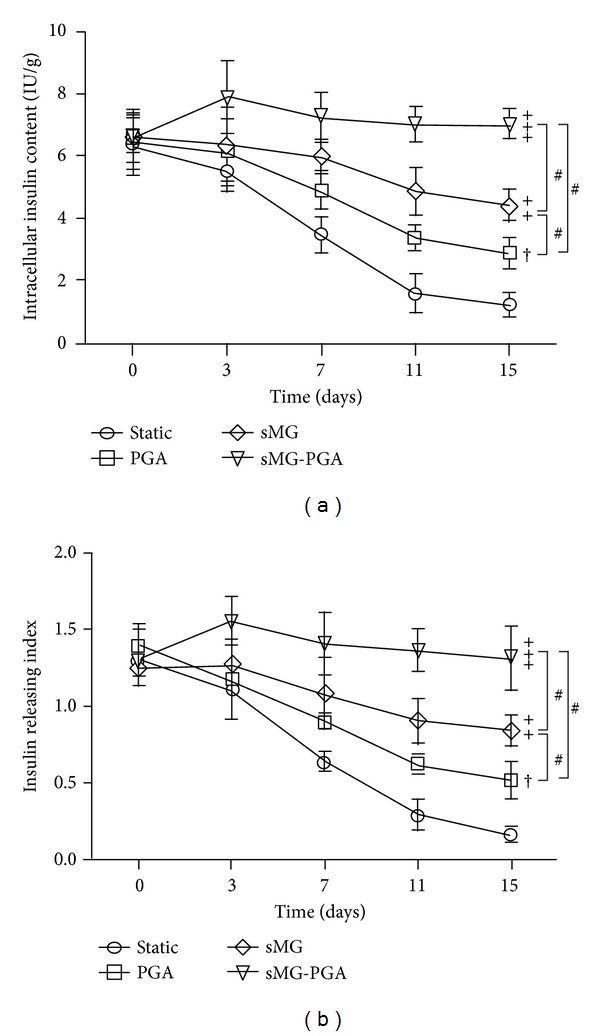
Assessment of the insulin production. The intracellular insulin content (a) and insulin-releasing index (b) were calculated to determine the functionality of insulin production. Significant increases are indicated by “†” at *P* < 0.05, “++” at *P* < 0.01, and “+++” at *P* < 0.001, compared with the static group. Significant differences between PGA and sMG and between PGA-sMG and PGA or sMG are denoted by “#.”

To evaluate the functionality of the islets, we examined their cellular insulin content and insulin-releasing indices. As shown in Figure 3(a), quantitative measurements of the intracellular insulin content were gathered to determine the secretory function of the cells under different conditions. At day 0, no measurement differences were observed among all groups. Compared with the static group, the cellular insulin content of cells grown under the PGA condition was not altered at day 3 but was 40% (*P* < 0.05) higher after day 7. At days 11 and 15, this alteration increased further, becoming more than 1-fold (*P* < 0.01) and nearly 1.5-fold (*P* < 0.01) higher in cells grown under the PGA condition as compared with the static condition. Additionally, at day 3, the amount of intracellular insulin of cells grown under the sMG condition was comparable with that of cells from the static group. Relative to the changes in the intracellular insulin content between cells grown under the PGA and static conditions, the differences between the sMG and static groups were more prominent, being 70% (*P* < 0.01), more than 2-fold (*P* < 0.001), and almost 2.5-fold (*P* < 0.001) higher in the sMG group at days 7, 11, and 15, respectively, compared with the matching static conditions. The PGA-sMG condition induced a slight upregulation of the intracellular insulin content as early as day 3, with the intracellular insulin contents of these cells being 25% higher than those of cells in the PGA and sMG groups (both *P* < 0.05) and 40% (*P* < 0.05) higher than those of cells in the static group. At days 7, 11, and 15, the cells cultured under the PGA-sMG conditions displayed more significant changes in their cellular insulin contents, being more than 1-fold (*P* < 0.01), nearly 3.5-fold (*P* < 0.001), and almost 5-fold (*P* < 0.001) higher, respectively, than those of cells grown under the matching static conditions. Among all the conditions except for static culture, the level of intracellular insulin increased in the PGA-sMG group by almost 50% (*P* < 0.05), more than 1-fold (*P* < 0.01), and 1.5-fold (*P* < 0.001) at days 7, 11, and 15, respectively, compared with the PGA group. The amount of cellular insulin under the PGA-sMG condition was more than 20% (*P* < 0.05), almost 50% (*P* < 0.05), and 75% (*P* < 0.05) higher than that in cells from the matching sMG group at days 7, 11, and 15, respectively. A difference between the PGA and sMG groups was also measured at days 11 and 15 (both *P* < 0.05).

Among the four groups, the differences in the insulin-releasing index were similar to the differences in the cellular insulin content at each time point (Figure 3). Briefly, the insulin-releasing index in PGA and sMG groups was higher than that of static culture group at days 7 (*P* < 0.05, *P* < 0.01), 11 (*P* < 0.01, *P* < 0.001), and 15 (*P* < 0.01, *P* < 0.001), respectively. In contrast, the cells cultured under the PGA-sMG conditions began to display an increased level of the insulin-releasing index from day 3 on *P* < 0.05 and demonstrated more prominent alterations in the insulin-releasing index at days 7 (*P* < 0.01), 11 (*P* < 0.001), and 15 (*P* < 0.001), respectively, as compared with the cells grown under the matching static conditions. Within all the conditions except for static culture, PGA-sMG culture displayed a higher level in the insulin-releasing index than the PGA and sMG conditions at days 3 (both *P* < 0.05), 7 (*P* < 0.05, *P* < 0.05), 11 (*P* < 0.01, *P* < 0.05), and 15 (*P* < 0.001, *P* < 0.05), respectively. Comparably, a measurable difference between the PGA and sMG groups with regard to insulin-releasing index was found at days 11 and 15 (both *P* < 0.05). 

### 3.3. Morphological Observation

**Figure 4:**
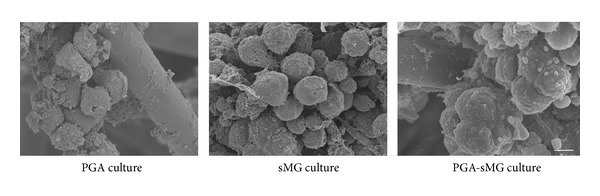
Morphological analysis of the islets under scanning electron microscopy (×1800) (bar = 50 *µ*m).

Morphological analysis under SEM of the islets at day 11 demonstrated that the PGA-sMG treatment preserved the integrity of the islets and facilitated the formation of tight intercellular and cells-scaffolds connections. In contrast, incomplete islets and loose intercellular connections were observed in both PGA and sMG groups (Figure 4).

## 4. Discussion

Islet transplantation, characterized as a superior alternative to the exogenous administration of insulin, is thought to potentially reverse the hyperglycemia observed in cases of diabetes [3]. However, limitations in the quantity and quality of isolated islets inevitably establish barriers to islet transplantation in diabetes treatment. PGA scaffolds, with their advantages of a suitable spatial structure and excellent biodegradability and biocompatibility, have been applied in a wide range of cell differentiation and tissue repair protocols [32, 33]. In addition, sMG has been proven to confer many benefits for cells in culture, such as lower hydrodynamic shear stress and the transfer of abundant nutrients [17, 18]. In this study, we explored whether PGA combined with sMG could serve as a preferable alternative method for islet culture *in vitro*.

In our recent study, PGA scaffold culture conditions were confirmed to promote cellular adhesion and viability by providing better nutrients and a suitable microenvironment [7]. We demonstrated that, due to the porous structure of the 3D scaffolds and the full infiltration of nutrients (Figure 1), the PGA treatment facilitated the tight adherence of pancreatic islets to the scaffold fibers and had the prominent effect of reversing the decrease in cell viability observed in the static culture condition (*P* < 0.05, Figure 2). This effect arose after day 7 and gradually increased as the time course of the treatment was extended. Similarly, the sMG condition also showed enhanced cell viability relative to the static culture, but the viability of cells cultured under the sMG condition did not differ from that of cells cultured under the PGA condition within a 15-day culture (Figure 2). This seeming similarity in cell viability between the two conditions was not sustained; the sMG culture demonstrated a preferential effect on the maintenance of cell survival in long-term culture as compared with the PGA culture (*P* < 0.05, see Figure S1 in the Supplementary Material available online at http://dx.doi.org/10.1155/2013/150739). This difference could be explained by the degradation of the PGA scaffolds, which resulted in islets becoming stacked due to the loss of support by the scaffolds and the loss of their 3D metabolic environments, in turn resulting in a decrease in cell viability. At day 15, the degradation of the PGA scaffolds could be detected, and the outer layers of the PGA scaffolds began to fall off under SEM (data not shown). In the sMG group, islets that continuously experienced the microgravity that contributes to the sufficient transfer of nutrients and waste excretion throughout the entire treatment process were maintained in good condition. With regard to the islets cultured under the static condition, their limited nutrient supply and accumulation of metabolic wastes failed to reverse their decreased viability, resulting in the worst survival rate across all studied conditions (Figure 2). As expected, the PGA-sMG group integrated the advantages of both methods to most significantly improve islet survival, even in long-term culture, as compared with the other three conditions (Figure 2, Figure S1). In the present study, we found that the degradation of the scaffolds in the PGA group was observed at day 15 but was delayed for 5 days in the PGA-sMG group. At day 25, the scaffolds were completely degraded in the PGA group, while the degraded fragments of the scaffolds were still observed in the PGA-sMG group (Figure S3). This difference of degradation rate between both groups may be due to the reduced pH values caused by the insufficient transfer of metabolic wastes that likely promoted the degradation of the scaffolds in the PGA group [34, 35], which in turn led to islet aggregation without the support of the scaffolds and decreased islet survival. Thus, the present study indicates that the PGA-sMG treatment significantly improves the viability of islets by bettering their metabolism and microenvironment.

Insulin production functionality is important in the evaluation of islets, especially isolated islets grown *in vitro*. In the present study, cells in both the PGA (*P* < 0.05) and sMG (*P* < 0.01) conditions significantly improved their insulin-producing activity after day 7, as demonstrated by examination of their cellular insulin content and insulin-releasing indices as compared with cells from the static group (Figure 3). Notably, the sMG treatment demonstrated the preferable effect of promoting insulin secretion after day 11 as compared with the PGA treatment (*P* < 0.05, Figure 3), but this difference could not be observed in the cell viability observed over the 15-day treatment (Figure 2). This discrepancy from the above result reveals that, during short-term culture, despite the lack of a significant improvement in islet survival between the PGA and sMG conditions, the sMG condition most likely improves the ability of the islets to promote insulin release, which may be attributed to the preferable dynamic features of sMG. Similarly, the PGA-sMG treatment significantly improved the insulin-producing function of the islets as early as day 3 as compared with any of the other conditions (Figure 3). Interestingly, the difference in cell viability between the PGA-sMG condition and the other groups appeared at day 7 (Figure 2). This asynchronization between cell viability and insulin production implies that at early stages the PGA-sMG condition promotes the improvement of insulin-producing activity prior to the improvement of islet viability. In addition, the insulin-producing activity of the islets displayed a slight upregulation under PGA-sMG treatment after 3 days, suggesting a role of the PGA-sMG condition in the fast improvement of the insulin-producing activity of the islets. The long-term *in vitro* culture of islets demonstrated that, although the viability of the islets in the PGA-sMG group was minimal at day 30, the insulin-releasing index in the PGA-sMG group reached almost 1.0, which was much higher than the insulin-releasing index of any other group. This indicates that PGA-sMG conditions play a strong and durable role in maintaining and improving the secretory function of islets that is likely due to the reduced production of molecules detrimental to the islets [21, 22] (Figure S1, Figure S2). 

## 5. Conclusions

Our data demonstrate that the PGA-sMG culture condition significantly improves the viability and secretory function and prolongs the survival time of isolated pancreatic islets. PGA scaffolds are endowed with favorable biocompatibility and biodegradability [32, 33] and provide a three-dimensional environment for the islets. Additionally, sMG provides sufficient transport of nutritional supplies and metabolic waste for the islets due to its characteristic dynamics [17, 18]. Thus, given the advantages of both PGA and sMG, the PGA-sMG culture condition likely provides a superior metabolic microenvironment for islets *in vitro*.

## Supplementary Material

FIGURE S1: Evaluation of islet viability in long-term culture. Among the four conditions, the static group demonstrated the worst survival rate in the long culture. The cells cultured in the sMG condition displayed a better effect on preserving islets survival than those in PGA. Comparably, the PGA-sMG treatment was proven to have a preferable ability to maintain the cell viability as compared with any one of the other three groups. Significant increases between PGA and sMG and between PGA-sMG and PGA or sMG are denoted by “#”.
FIGURE S2: Assessment of the functionality of insulin production of cells in long-term culture. Compared with the other three groups, the static condition undoubtedly failed to reverse decreased insulin secretion. The sMG group had a preferable role in improving the secretory function of islets than the PGA group. The cells in the PGA-sMG condition displayed the strongest ability to promote insulin production across all studied conditions. Significant increases between PGA and sMG and between PGA-sMG and PGA or sMG are denoted by “#”.
FIGURE S3: Observable biodegradability of PGA scaffolds at days 25 in PGA-sMG culture condition under scanning electron microscopy (×1800) (bar = 50 *µ*m). The degraded fragments of the scaffolds were visualized in the PGA-sMG group under SEM.
