# Supplementary material for: Simulated Microgravity Combined with Polyglycolic Acid Scaffold Culture Conditions Improves the Function of Pancreatic Islets
Source: Biomed Res Int. 2013 Aug 6;2013:150739. doi: 10.1155/2013/150739 (PMC3758870; doi:10.1155/2013/150739)
Supplement: Supplementary file 1 — FIGURE S1: Evaluation of islet viability in long-term culture. Among the four conditions, the static group demonstrated the worst survival rate in the long culture. The cells cultured in the sMG condition displayed a better effect on preserving islets survival than those in PGA. Comparably, the PGA-sMG treatment was proven to have a preferable ability to maintain the cell viability as compared with any one of the other three groups. Significant increases between PGA and sMG and between PGA-sMG and PGA or sMG are denoted by “#”. FIGURE S2: Assessment of the functionality of insulin production of cells in long-term culture. Compared with the other three groups, the static condition undoubtedly failed to reverse decreased insulin secretion. The sMG group had a preferable role in improving the secretory function of islets than the PGA group. The cells in the PGA-sMG condition displayed the strongest ability to promote insulin production across all studied conditions. Significant increases between PGA and sMG and between PGA-sMG and PGA or sMG are denoted by “#”. FIGURE S3: Observable biodegradability of PGA scaffolds at days 25 in PGA-sMG culture condition under scanning electron microscopy (×1800) (bar = 50 µm). The degraded fragments of the scaffolds were visualized in the PGA-sMG group under SEM. [file 150739.f1.doc]

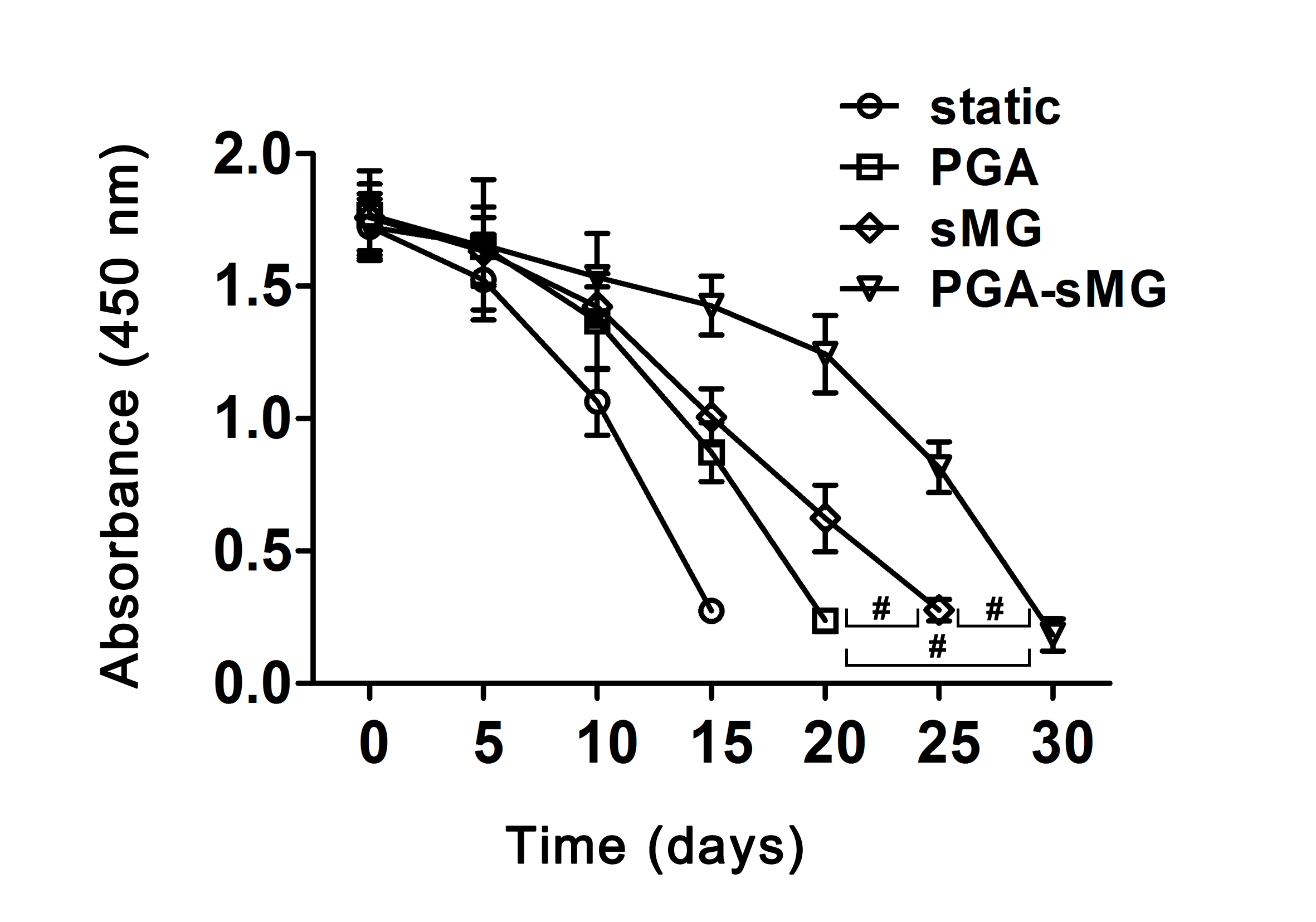


FIGURE S1: Evaluation of islet viability in long-term culture. Among the four conditions, the static group demonstrated the worst survival rate in the long culture. The cells cultured in the sMG condition displayed a better effect on preserving islets survival than those in PGA. Comparably, the PGA-sMG treatment was proven to have a preferable ability to maintain the cell viability as compared with any one of the other three groups. Significant increases between PGA and sMG and between PGA-sMG and PGA or sMG are denoted by “#”.


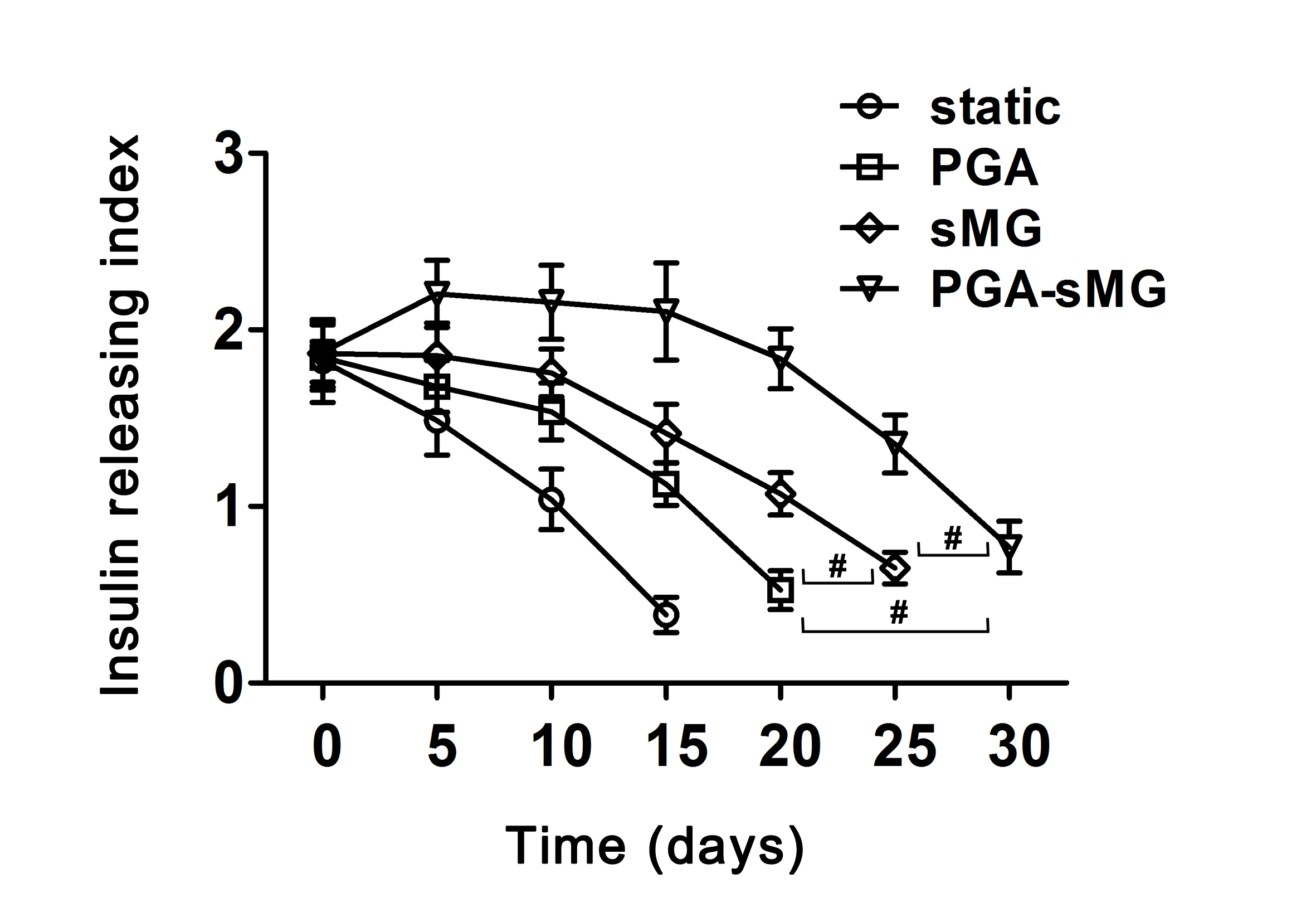


FIGURE S2: Assessment of the functionality of insulin production of cells in long-term culture. Compared with the other three groups, the static condition undoubtedly failed to reverse decreased insulin secretion. The sMG group had a preferable role in improving the secretory function of islets than the PGA group. The cells in the PGA-sMG condition displayed the strongest ability to promote insulin production across all studied conditions. Significant increases between PGA and sMG and between PGA-sMG and PGA or sMG are denoted by “#”.


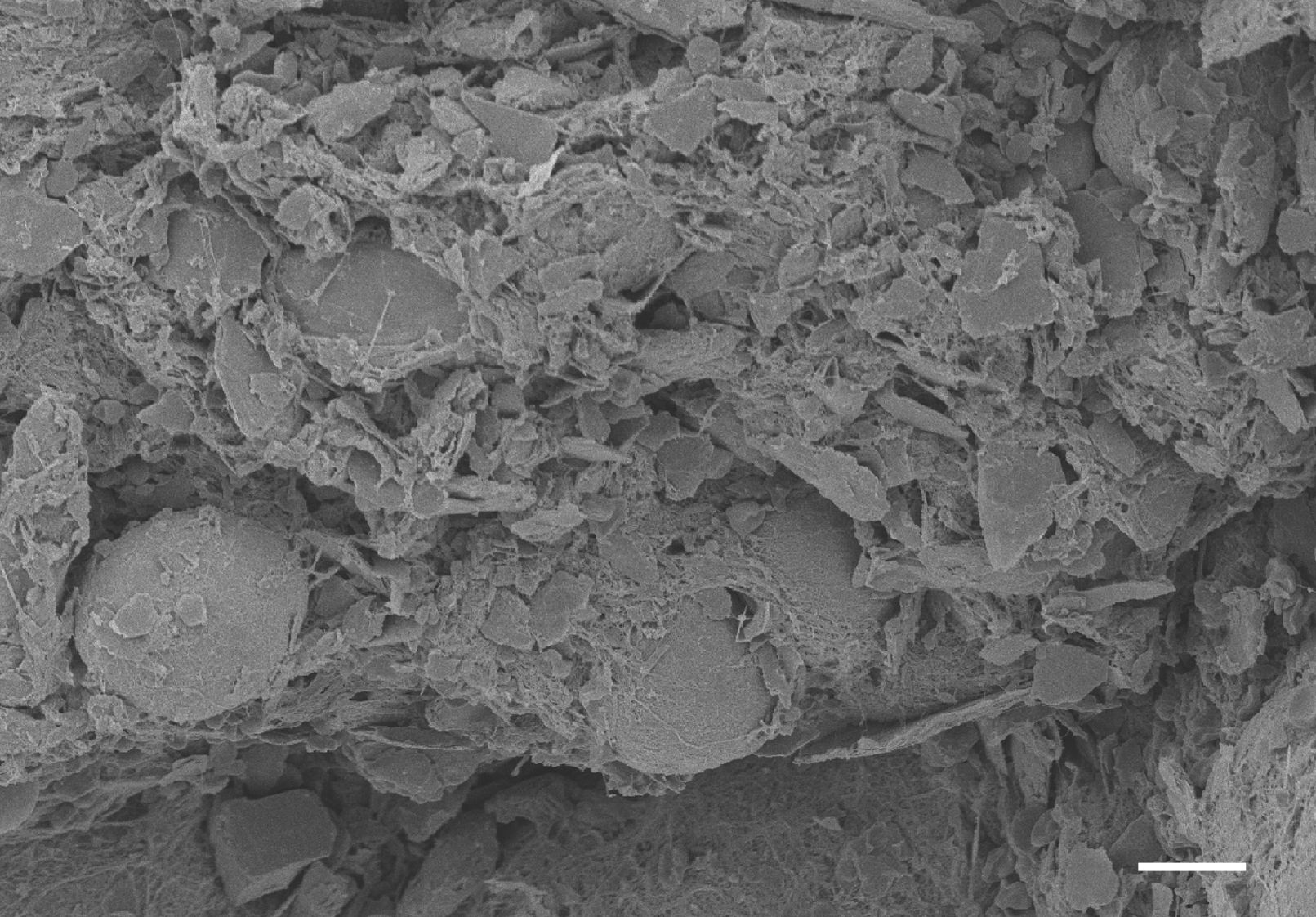


FIGURE S3: Observable biodegradability of PGA scaffolds at days 25 in PGA-sMG culture condition under scanning electron microscopy (×1800) (bar = 50 µm). The degraded fragments of the scaffolds were visualized in the PGA-sMG group under SEM.
